# Azolla mediated alterations in grain yield and quality in Rice

**DOI:** 10.1111/ppl.70158

**Published:** 2025-03-26

**Authors:** Nadia Bazihizina, Chiara Paleni, Stefania Caparrotta, Tania Macchiavelli, Giorgia Guardigli, Ilaria Colzi, Michele Petrillo, Cristina Gonnelli, Antonietta Saccomanno, Veronica Gregis, Stefano Mancuso, Diego Comparini, Martin M. Kater, Camilla Pandolfi

**Affiliations:** ^1^ Department of Biology Università degli Studi di Firenze Florence Italy; ^2^ Department of Biosciences Università degli Studi di Milano Milan Italy; ^3^ Department of Agriculture, Food, Environment and Forestry Università degli Studi di Firenze Sesto Fiorentino Italy

## Abstract

Rice is one of the most important cereal crops worldwide. To boost its production in a sustainable manner, co‐cultivation with *Azolla* species is often used to supplement its nitrogen (N) demands. However, beyond N nutrition, the physiological and developmental effects of azolla on rice remain unclear. This study investigates these mechanisms by analysing growth, inflorescence meristem transcriptomics, yield, and grain ionomics in rice plants grown alone (R) or with azolla (R + A) in non‐limiting N conditions. During the vegetative stage, the presence of azolla increased allocation of resources to rice shoots without affecting root growth, while in the reproductive stage, it improved panicle architecture, with a 6% increase in length and up to 26% increase in panicle branching. Nevertheless, while this increase in panicle branching in R + A translated into a greater number of grains per plant, grain weight declined. As a result, yields were similar between R and R + A. There was also an azolla‐induced increment in several mineral elements in R + A grains, with the notable exception of zinc, which declined by more than 30%. Finally, the presence of azolla altered the expression of several gene families, and in particular, it led to the upregulation of numerous transcription factors from the AP2/ERF, WRKY and NAM families. Interestingly, the presence of azolla also led to the upregulation of several genes (including WRKY transcription factors) involved in resistance to several pathogens and abiotic stresses. Overall, our results suggest that rice‐azolla co‐cultivation has implications that go beyond N‐nutrition for sustainable intensification of rice production.

## 1 INTRODUCTION

Plants are the primary source of food for humans. The world population is expected to touch 10 billion people in 2050 from the current 7.9 billion, emphasising the need for a proportional increase in food production. This is especially relevant for the most important staple crops (Ramankutty et al. 2018). Amongst these, rice is one of the most important cereal crops worldwide and is a staple food source for more than half of the global population, providing 20% of the world's dietary energy supply, especially in Asia and Africa (FAO 2018, Fukagawa and Ziska 2019; Li et al. 2021). While production will have to double by 2050 relative to 2010, insufficient availability of arable land, increasing yield fluctuations, and the changing climate are and will further hamper our ability to meet this goal while remaining environmentally sustainable (Li et al. 2021). In this context, nature‐based solutions can provide ecologically effective and environmentally friendly approaches to sustainably increase rice production to cater for the growing food demands. One of these solutions is the use of co‐cultivation of rice with *Azolla* species, which are floating freshwater ferns that form a permanent, hereditary symbiosis with a nitrogen‐fixing, heterocyst‐forming cyanobacterium, *Trichormus azollae*, in their leaf cavities (Van Hove and Lejeune 2002; Kumar et al. 2019). The co‐cultivation of *Azolla* spp. has been commonly used as an organic nitrogen fertiliser (with azolla provided as green manure or in co‐cultivation with rice) in parts of Asia, especially in Vietnam and southern China, with positive outcomes in terms of rice crop productivity (Kröck et al. 1988; Vaishampayan et al. 2001; De Macale and Vlek 2004; Bhuvaneshwari and Singh 2015; Marzouk et al. 2023; Oyange et al. 2019). The presence of azolla has been reported to have either a neutral or positive effect on grain yield in conditions where N was limited, with few reports indicating an increase in tiller number and grain number per panicle (Kröck et al. 1988; De Macale and Vlek 2004; Bhuvaneshwari and Singh 2015; Oyange et al. 2019). This has been linked with the improved N supply and conservation in the system, facilitated by the biological N fixation by the symbiont and the reduction in NH_3_‐volatilization (Vlek et al. 1996; De Macale and Vlek 2004). However, it remains unclear whether and how azolla could improve rice development beyond its effect in improving N nutrition and whether additional azolla‐mediated processes could be used to optimise rice production systems, improving crop yield and/or grain quality.

The three main morphological traits defining rice productivity are panicle number, grain weight (determined by grain size and filling) and grain number per panicle (affected by panicle branching) (Xing and Zhang 2010; Wang and Li 2019; Li et al. 2021; Khush, 1995; Yan et al., 1998). Grain number per panicle is a multifactorial trait primarily determined during the early stages of reproductive development, when primary and secondary branch meristems and spikelet meristems are formed (Tanaka et al. 2013; Caselli et al. 2020). The inflorescence produces grains from fertilised florets, and their number depends on the numbers and structures of branches and florets within each inflorescence, thus determining rice reproductive success and overall yield. This developmental progression is a finely tuned process regulated by different molecular pathways controlling inflorescence meristem activity in response to environmental/external and endogenous signals (Zhang and Xie 2014; Wang et al. 2019; Chun et al. 2022; Sreenivasulu and Schnurbusch, 2012). While many genes have been reported to influence panicle architecture in rice (Li et al. 2021; Guo et al. 2023), transcription factor encoding genes are known to be key players in this process (Wei et al. 2022). Important transcription factor genes in this respect belong to the *ALOG*, MADS‐box and AP2/ERF families (Jisha et al. 2015; Xie et al. 2022; Zhu et al. 2022). For instance, loss‐of‐function mutations in *ALOG* genes have been shown to reduce branching, whereas overexpression caused an opposite phenotype, increasing branches and, thereby, seed number (Yoshida et al. 2013, Beretta et al. 2023).

Here, we investigated in detail the impact of azolla‐rice co‐cultivation on rice physiology and yield attributes. As mentioned above, aside from the increase in root‐zone N availability, very little is known about other potential key mechanisms through which the beneficial properties of the azolla on rice are realised. Thus, to delve deeper into the mechanisms underlying improved performance with azolla co‐cultivation, we analysed the physiological response during the vegetative phase and panicle morphology and grain number in rice plants grown with and without azolla in non‐limiting N conditions. Furthermore, to pin down the mechanism underlying eventual changes in panicle morphology and grain number, we performed an RNA sequencing and differential gene expression analysis using inflorescence meristem transcriptomes. Overall, our study demonstrates that the presence of azolla, despite having no overall effect on crop yield in a non‐limiting N condition, improved panicle architecture and altered grain quality and attributes. In particular, the co‐cultivation enhanced the concentration of several mineral elements, with the important exception of the micronutrient Zn. RNA‐seq further revealed key association patterns linked to the presence of azolla and its symbiotic cyanobacteria, which is important for understanding possible alterations in concerted molecular networks controlling panicle architecture.

## 2 MATERIALS AND METHODS

### 2.1 Growing conditions

To assess how the presence of azolla affected rice performance in the azolla‐rice co‐cultivation, we grew *Oryza sativa* sp. *japonica* cv. Kitaake, an early‐flowering rice cultivar, with the aquatic fern *Azolla filiculoides*. Three rounds of experiments were conducted in a growth chamber with a temperature between 26–30°C, relative humidity between 60–70%, and a 14/10 h day/night cycle, 500–1000 μmol m^−2^ s^−1^ of photosynthetically active radiation (PAR), (C‐LED Toplight Plus, Italy). All experiments tested two growing systems: (1) rice grown alone, considered as the control treatment (R) and (2) rice co‐cultivated with azolla (R + A). Rice plants were grown in a Yoshida solution (Yoshida et al. 1971; Gregorio et al. 2000) adjusted with a 1 M KOH at pH 5.5. To prevent pH change 2‐N‐morpholino‐ethanesulfonic acid (MES) hydrate was added with a final concentration of 5 mM (Ali et al. 2020). The nutrient solution for hydroponic rice cultivation was renewed weekly by siphoning it out with a tube placed at the bottom of the container and refilling it with a funnel to avoid disturbing the azolla. Changing the solutions removed the dead/decomposed azolla plants; thus, when needed, new azolla plants were added to ensure the full coverage of the surface. Frequent replacement of the growth media and the use of actively growing Azolla ensured that the inorganic N available to rice plants in both treatments (R and R + A) remained similar. This approach, as demonstrated by Cannavò et al. (2024), ensured that the study focused exclusively on the effects induced by azolla beyond N nutrition.

### 2.2 Plant growth

Azolla was bought from a local nursery in Pistoia (Italy) and further identified as *Azolla filiculoides* (Lam.) on the basis of both molecular and morphological markers (Costarelli et al. 2021). Before being transferred into the pots with rice plants, the aquatic fern was propagated using the Watanabe nutrient solution (Watanabe 1992), and then, 1 week prior to the co‐cultivation, the azolla was transferred into the Yoshida nutrient solution. Microscopic detection of the symbiont and auxin quantifications using the Salkowski reagent method (Guardado‐Fierros et al. 2024) were conducted to ensure that cultivation in the Yoshida nutrient solution, given the high N concentrations, did not influence the symbiont (Figure S1).

Rice seeds were surface sterilised in sodium hypochlorite solution (0.05%) for 1 min and washed three times with distilled water. To promote synchronous germination, rice seeds were distributed on rolls of absorbent paper and inserted in jars filled with distilled water to ensure constant hydration and placed in a small germination chamber in darkness at a constant temperature of 28°C. After 3 days, seedlings were transferred to the growth chamber described above and placed in 25% Yoshida solution. Uniform seedlings (hypocotyl height of about 6 cm) were then selected and transferred into a floating mesh placed in aerated 50% Yoshida solution for 4 additional days. Seedlings were then transferred in a hydroponic setup where seedlings were placed in 5.5 cm mesh pots filled with expanded clay with a full‐strength nutrient solution (7 days after germination, 2 leaf stage). Six mesh pots were anchored to 3 L containers and covered with a mesh to prevent the escape of the expanded clay once immersed in the solution. In the rice‐azolla co‐cultivation, azolla plants were then added to cover the surface.

### 2.3 Experiment to evaluate impact on growth and physiology during the vegetative stage

In a first experiment to evaluate the impact of azolla on rice growth and physiology, plants (for each treatment, *n* = 8) were grown as described above and harvested 30 and 60 days after adding the azolla in the co‐cultivation treatment and shoot and root fresh (FW) and dry weight (DW) determined. At each harvest, the number of tillers and leaves per plant were counted. Leaf area was also measured 60 days after adding azolla using a scanner (300 dpi resolution), and the total leaf area (cm^2^) was calculated using Image‐J software. To obtain the dry weight, plant material was inserted in a 50°C oven for about one week until a constant weight was achieved.

Leaf pigment concentrations were determined using a slightly modified protocol (Welburn 1994; Atzori and Caparrotta 2023; Bazihizina et al. 2024). Briefly, about 0.1 g of fresh leaves were collected, weighed, snap‐frozen in liquid nitrogen and stored at −80°C. Then, a tungsten bead (ø 5 mm) was added to each centrifugation tube, and plant tissue was mechanically disrupted using the Tissue Lyser II system (QIAGEN, cat. no. 85,300) for 30 s at 30 Hz. Subsequently, a pure methanol solution at 4°C was used to extract pigments and samples were incubated in darkness at 4°C for 30 min. Subsequently, samples were centrifuged for 10 min at about 5000 *g* at 4°C, the supernatant collected, and absorbance taken at 470, 665.2, and 652.4 nm using a multi‐reader (Tecan Infinite 200 Spectrophotometer) by inserting 200 μL in a transparent 96‐well microplate. Chlorophyll *a* (Chl *a*) and *b* (Chl *b*), as well as total carotenoid concentrations, were quantified using the equations described by Wellburn (1994) and normalised on the fresh weight (FW).

The portable photosynthesis system Li‐6400 XT (LiCor Inc.) was used to measure leaf gas exchanges using a Li‐6400‐40 leaf chamber (Li‐Cor) on the youngest fully expanded leaves at the end of the 60‐day cultivation period. Measurements of net photosynthetic rate (A_n_) and stomatal conductance (g_s_) were determined with a reference CO_2_ of 400 μmol mol^−1^, ambient relative humidity, flow rate of 500 μmol s^−1^, chamber temperature at 28°C and 1000 μmol m^−2^ s^−1^ of PAR.

### 2.4 Experiment to assess impact on rice yield and phenotypic analysis of panicles and grains

A second experiment was conducted to evaluate how the presence of azolla affected rice yield and its attributes (for each treatment, *n* = 12). The growth conditions and setup used were as described above. Plants were harvested 120 days after the beginning of the co‐cultivation when the percentage of yellow‐coloured grains on the panicle was 80–85% (Atapattu et al. 2018). As no differences were seen between R and R + A plants, both were harvested at the same time. All whole plants were hung to dry for approximately two weeks with a ventilation fan (Atungulu and Sadaka 2019). The phenotypic analysis was performed on the panicle from the main tiller of each plant. Each panicle was attached to a white paper, and all panicle branches were spread and blocked with transparent tape and scanned. Finally, 100 grains from all panicles per plant in each treatment were randomly collected and scanned. The images were analysed using the ImageJ software, with the plugin “SeedsAnalyzer” for the grain analysis (Loddo et al. 2023). The percentage of ripened grains (filled vs. unfilled grains) was estimated using the method described in Kumar et al. 2021. Briefly, to measure the ripening rate in R and R + A plants, the separated grains were immersed in a NaCl solution with a specific gravity of 1.06 (prepared by dissolving 90 g of NaCl in one L of distilled water). Then, the solution was stirred, and all grains floating or settled at the bottom were counted. Floating grains were categorized as unfilled, while those that sank were categorized as filled.

Ground dry grains were then oven‐dried at 60°C for five days and the element concentrations were analysed with a Z‐Spec E‐maxULTRA instrument (Z‐Spec Inc.) as in Bettarini et al. (2024). In short, the instrument uses monochromatic X‐ray fluorescence analysis technology (HDXRF technology) to analyze elements with Z = 15 (P) to Z = 39 (Y) on the K‐lines and up to Z = 92 (U) on the L‐lines. Samples were analyzed for 60 s in plant mode. Triplicate measurements were obtained from each sample at different locations within the sample to account for potential heterogeneity. The average of the three measurements was then used for further analysis. The validity of the analysis was confirmed using certified reference materials (e.g., ERM‐CD281, NIST 1573a, NIST 1570a, NIST 1567b, NIST 1568b, NMIJ 7502a, ERM BD151 standards).

### 2.5 Experiments to assess impact on reproductive meristem RNA‐seq

For the collection of the floral meristem at the correct stage, a third round of experiments was conducted. Here, several batches of plants were grown 2 weeks apart to characterise the meristem growth stage and infer the correct sampling time for the reproductive meristematic tissues, which was determined to be 45 days after germination. Then, plants were grown as described above, and inflorescence meristematic tissues were collected from the main stem during the transition from the primary branch meristem (PBM) to the secondary branch meristem (SBM) developmental stage, immediately frozen in liquid nitrogen and then stored at −80°C. For each of the two growth conditions (R, R + A), 3 replicates consisting of a pool of 4 rice inflorescence meristems each were then ground using a metal handheld tool for grinding directly in a 1.5 mL tube in liquid nitrogen. Total RNA was isolated using the RNeasy Plant Mini Kit (PMK) (Qiagen) from approximately 100–120 mg of ground tissue according to the manufacturer's instructions with a few modifications; DNase treatment was done using the Qiagen RNase‐free DNase set for the digestion of possible presence of DNA. The purity estimation of RNA was determined by ratios of *A*_260_/*A*_280_ and *A*_260_/*A*_230_ and quantified using Nanodrop (Nanodrop Lite Spectrophotometer, Thermo Scientific). The integrity of the RNA was evaluated by mini gel electrophoresis of RNAs for low amounts (less than 2 μg) by a standard, non‐denaturing 2% agarose gel and assayed by Novogene using the Agilent Bioanalyzer 5400 (Agilent Technologies). Stranded mRNA libraries were prepared with poly‐T oligo‐attached magnetic beads, followed by random hexamer priming and second‐strand cDNA synthesis with dUTP. Library preparation was created using 30 million pair‐end reads, PE150 strategy (Illumina) sequencing with a read length of 250 ~ 300 bp insert strand‐specific library by Novogene Europe service. Raw reads for the 3 replicates of each condition (R, R + A) were filtered to eliminate reads with adapter contamination, more than 10% uncalled bases, or more than 50% Q < 5 bases, and finally aligned to the reference genome with HISAT2 (Kim et al. 2019) by Novogene. Read counts were imported in R v4.0.2, and differential gene expression analysis between growth conditions was performed with DeSeq2 v1.28.1 (Love et al., 2014). To assess differences between the transcriptional profiles of co‐cultivated and control plants, a Principal Component Analysis (PCA) of the samples was plotted on the variance stabilizing‐transformed count data with the DESeq2 vst and plotPCA functions (Figure S4). Finally, the list of DE genes with criteria |logFC|>0, padj<0.05 was selected to run functional enrichment analysis on ShinyGO v0.80 (Ge et al. 2020) and CARMO (Wang et al. 2015), using the set of terms from Gene Ontology (Biological project, Molecular Function and Cellular Component) and KEGG, and using the list of all genes with detectable expression as background.

### 2.6 Statistical analysis

For plant growth, physiological performance, grain yield and ionomics, statistical analyses were performed using the software GraphPad Prism (GraphPad Software, www.graphpad.com), using the t‐test or one‐way analysis of variance (ANOVA), with a post‐hoc Tukey multiple comparison test to determine significant differences among means at *p* ≤ 0.05, depending on the dataset analysed.

## 3 RESULTS

### 3.1 Plant performances during the vegetative phase

**FIGURE 1:**
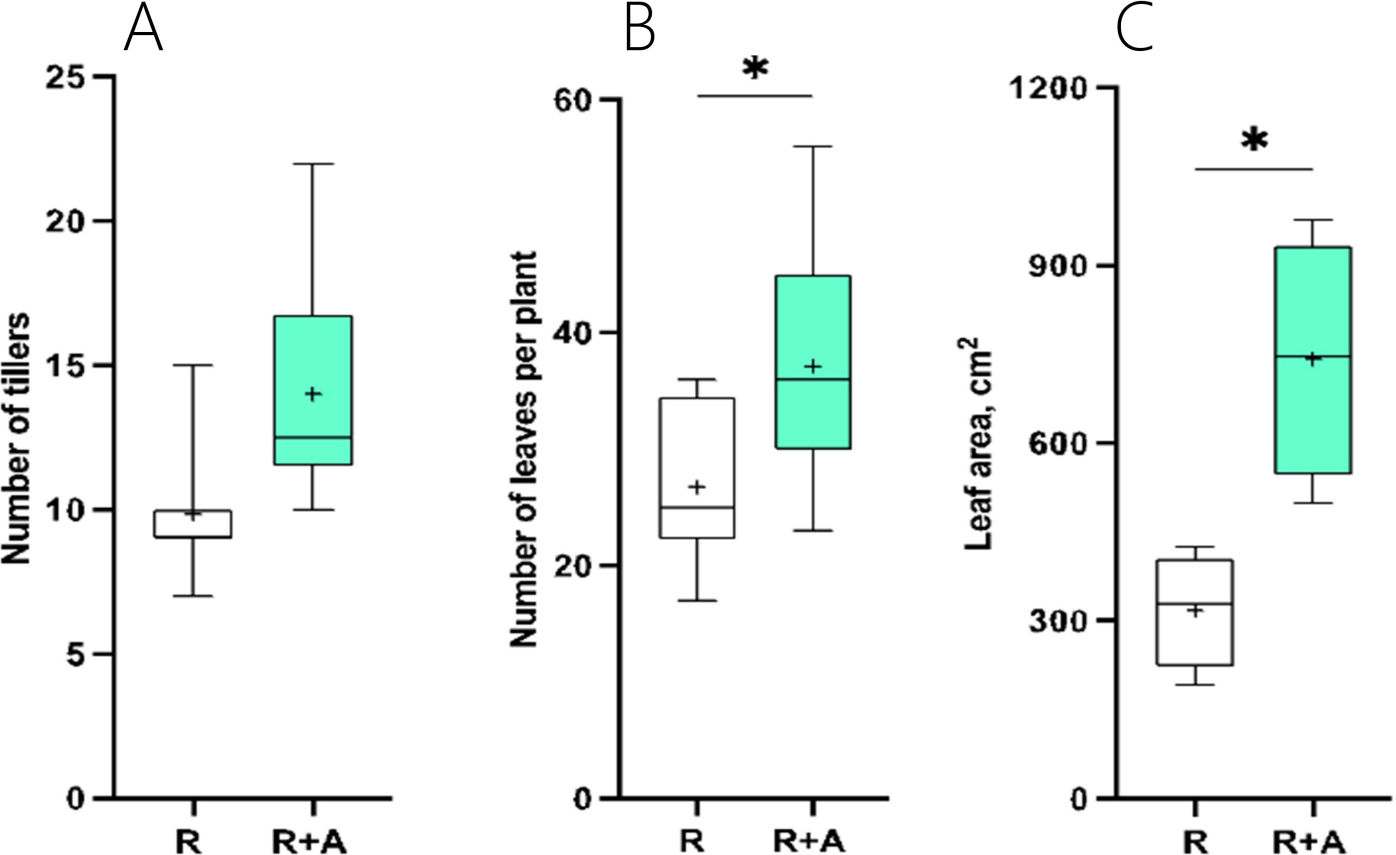
Boxplot graphs illustrating the effect of azolla on rice growth after 60 days of cultivation. R indicates rice grown without azolla, while R + A refers to rice plants grown in co‐cultivation with azolla. (A) number of tillers per plant; (B) average number of leaves per plant; (C) average leaf area. Asterisks denote significance levels from ANOVA analysis: *p < 0.05. *n* = 8.

**FIGURE 2:**
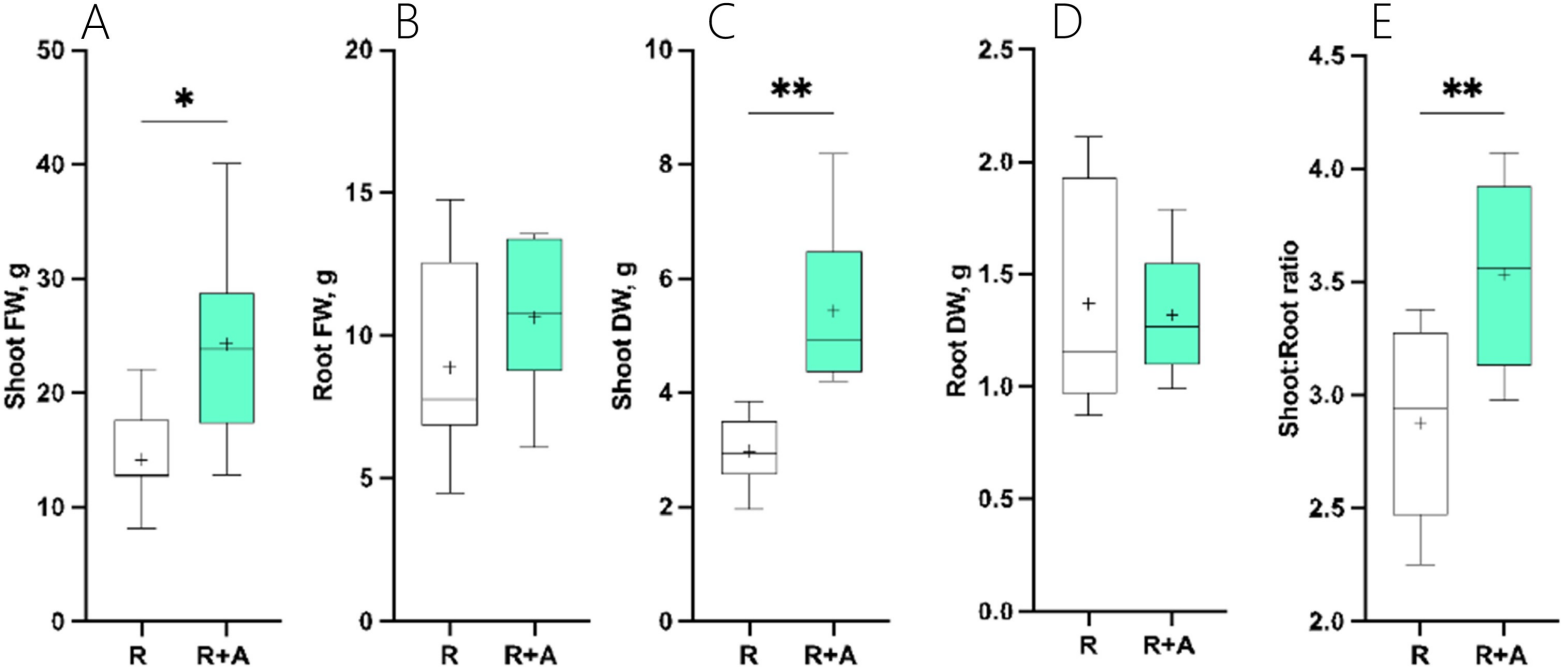
Boxplot graphs illustrating the effect of azolla on rice biomass production after 60 days of cultivation. (A) Shoot Fresh Weight, (B) Root Fresh Weight, (C) Shoot Dry Weight, (D) Root Dry Weight, (E) Shoot to root ratio. R indicates rice grown without azolla, while R + A refers to rice plants grown in co‐cultivation with azolla. Asterisks denote significance levels from ANOVA analysis: *p < 0.05, **p < 0.01. n = 8.

To study the effects that azolla has on rice growth and development in the co‐cultivation treatment (R + A) compared to rice plants growing without azolla (R), we analysed the number of tillers, the number of leaves and the leaf area. While no differences were seen after 30 days of co‐cultivation with azolla (Figure S2), significant differences emerged after 60 days of co‐cultivation. Indeed, while no significant differences were observed in the number of tillers per plant (Figure 1A, S2), after 60 days of cultivation, the presence of azolla led to a 1.4‐ to 2.3‐fold increase in the leaf number and leaf area per plant, respectively (Figures 1B, C, S2). This translated into a significant increase in both shoot fresh and dry mass (Figure 2A and C) in R + A plants compared to controls, while root fresh and dry mass remained similar to controls (Figure 2B and D). This relative increment in shoot biomass led to a 23% increase in shoot:root ratio compared to controls (Figure 2E). In parallel to rice growth, gas exchanges and leaf pigment concentrations were also monitored after 2 months of cultivation. As indicated in Figure S3, the presence of azolla in the co‐cultivation treatment had no overall effect on leaf pigment concentrations and gas exchanges when comparing R + A and R plants.

### 3.2 Yield analysis

**FIGURE 3:**
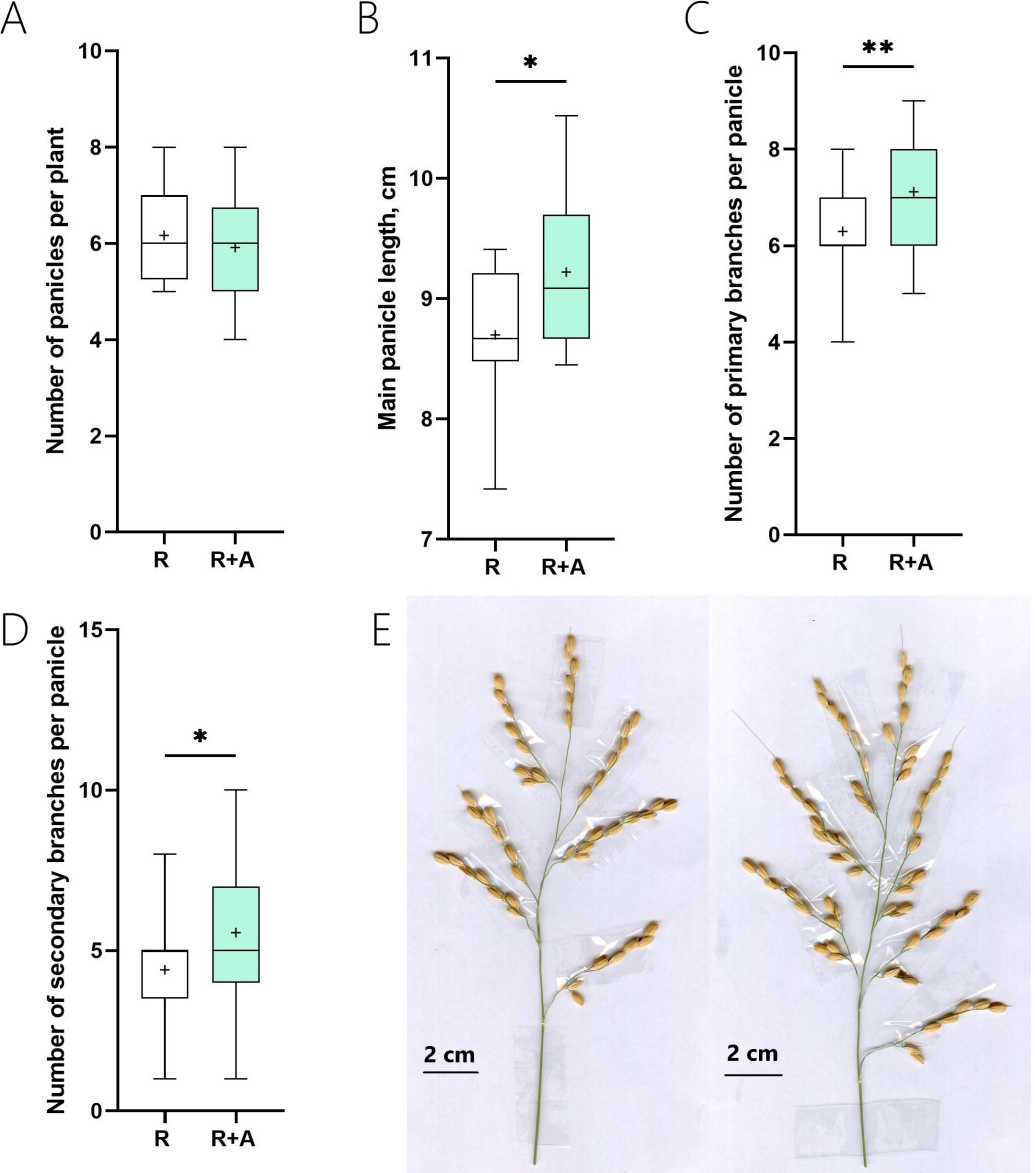
Boxplot graphs illustrating the effect of azolla on rice yield parameters. (A) Number of panicles per plant, (B) Main panicle length, (C) Number of primary branches per panicle, (D) Number of secondary branches per panicle, (E) Representative photo of the panicle of a rice plant grown without azolla and of a rice plant grown with azolla. R indicates rice grown without azolla, while R + A refers to rice plants grown in co‐cultivation with azolla. Asterisks denote significance levels from ANOVA analysis: *p < 0.05, **p < 0.01, ***p < 0.001, (*n* = 12).

**FIGURE 4:**
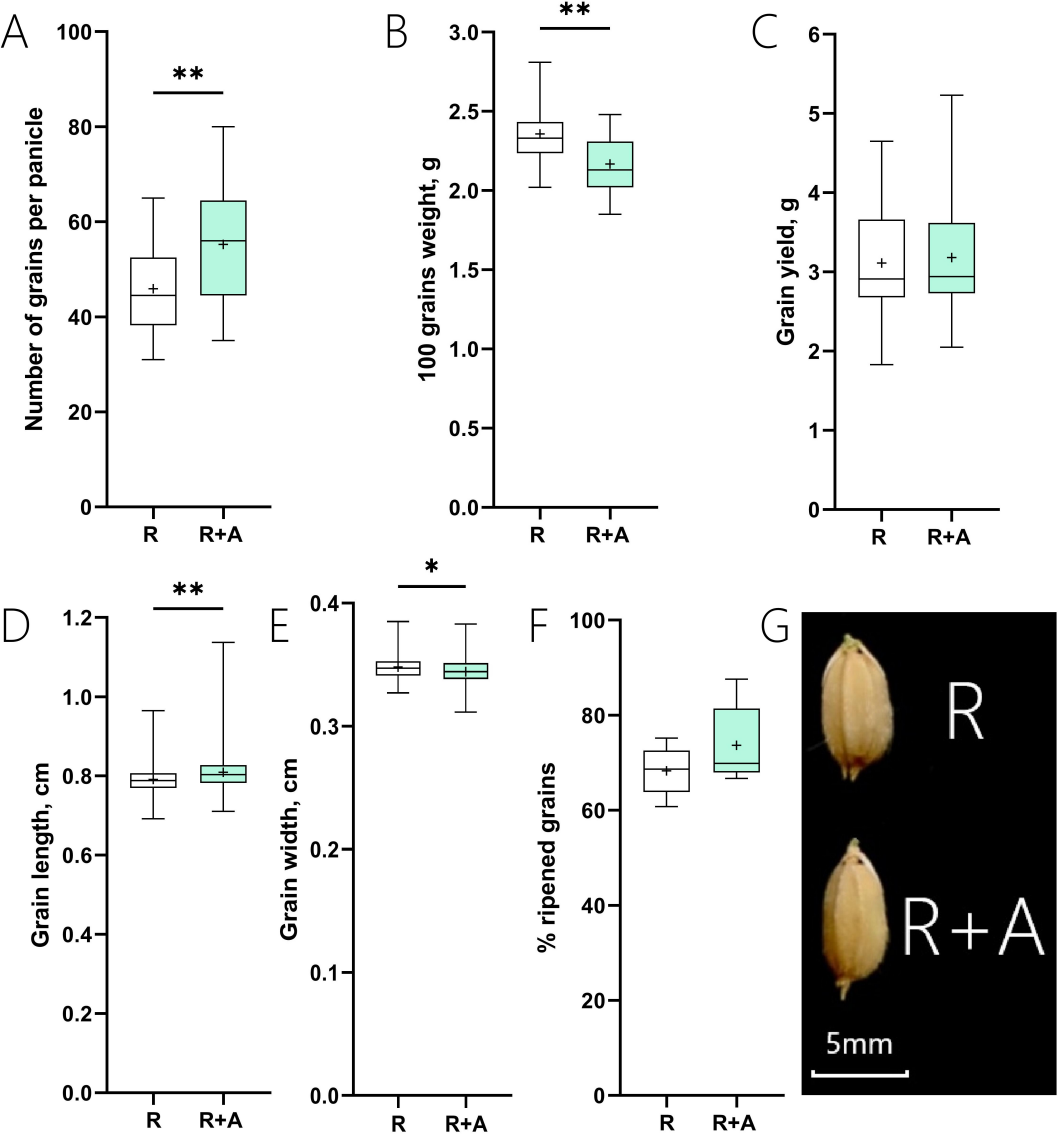
Boxplot graphs illustrating the effect of azolla on grain characteristics. (A) Number of grains per plant, (n = 12); (B) Weight of 100 grains, n = 12; (C) Grain yield, n = 12; (D) Grain length, *n* > 100; (E) Grain width, n > 100; (F) % ripened grains; and (G) Representative picture of grains produced by plants grown with and without azolla. R indicates rice grown without azolla, while R + A refers to rice plants grown in co‐cultivation with azolla. Asterisks denote significance levels from ANOVA analysis: *p < 0.05, **p < 0.01, ***p < 0.001.

Figure 3 shows that while the number of panicles per plant (Figure 3A) was not affected by the treatment, the presence of azolla in R + A plants led to an average 6% increase in panicle length (Figure 3B). The analysis of individual panicles also indicated that panicles in R + A had more branches, with on average 13 and 26% increases in the mean values for primary and secondary branches, respectively (Figure 3C, D, E). As a result of the increase in panicle branching, rice plants grown with azolla had a greater number of grains per panicle (an average 9 grains increase per panicle in R + A when compared to R, Figure 4A). An increase in grain length in R + A samples was also observed (on average 2% increase in R + A when compared to R, Figure 4D). Nevertheless, in R + A grown plants, there was a decline in the grain width, and the 100‐grain weight was declined by 8% when compared to R plants (Figure 4B, E). Consequently, despite the increased number of grains, the overall grain yield (weight) between R and R + A was not significantly different (Figure 4C). Similarly, the presence of azolla did not affect the ripening rates of the rice grains (Figure 4F).

**FIGURE 5:**
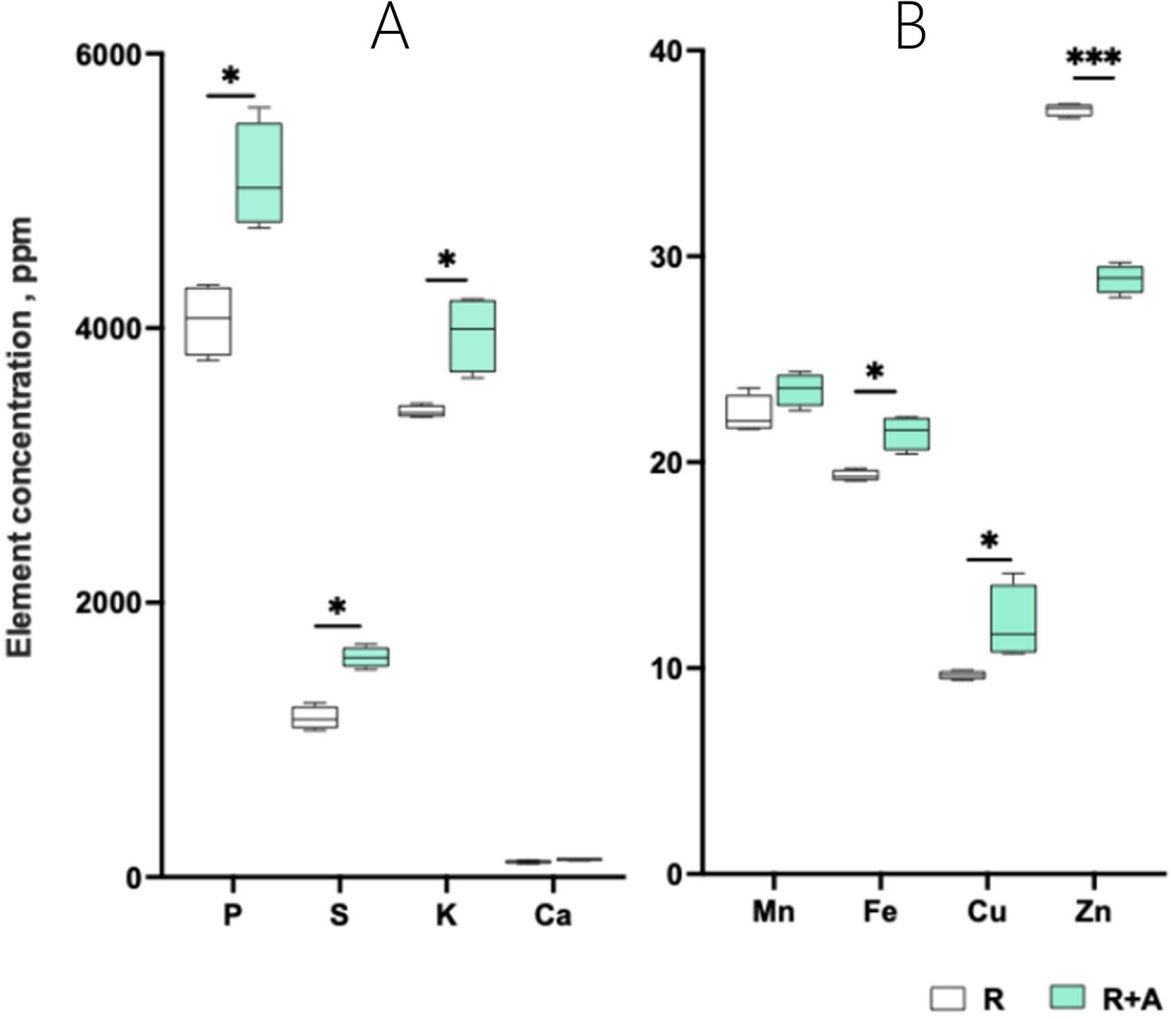
Boxplot graphs illustrating the effect of azolla on mineral element concentrations in rice grains. (A) P, S, K and Ca concentrations in rice grains; (B) Mn, Fe, Cu and Zn concentrations in rice grains. R indicates rice grown by itself, while R + A refers to rice plants grown in co‐cultivation with azolla. Asterisks denote significance levels from ANOVA analysis: *p < 0.05, **p < 0.01, ***p < 0.001. *n* = 4.

We also investigated whether the presence of azolla altered mineral element concentrations in grains. In general, for several elements, there was a significant increase (16 to 38%) in grains harvested from R + A plants when compared to R plants (Figure 5A, B), with significant increases for P, S, K, Fe and Cu. No significant differences between grains from R and R + A plants were observed for Ca and Mn, while Zn grain concentrations declined by an average of 33% in R + A compared to R grains.

### 3.3 RNA‐seq analysis during early stages of inflorescence development

**FIGURE 6:**
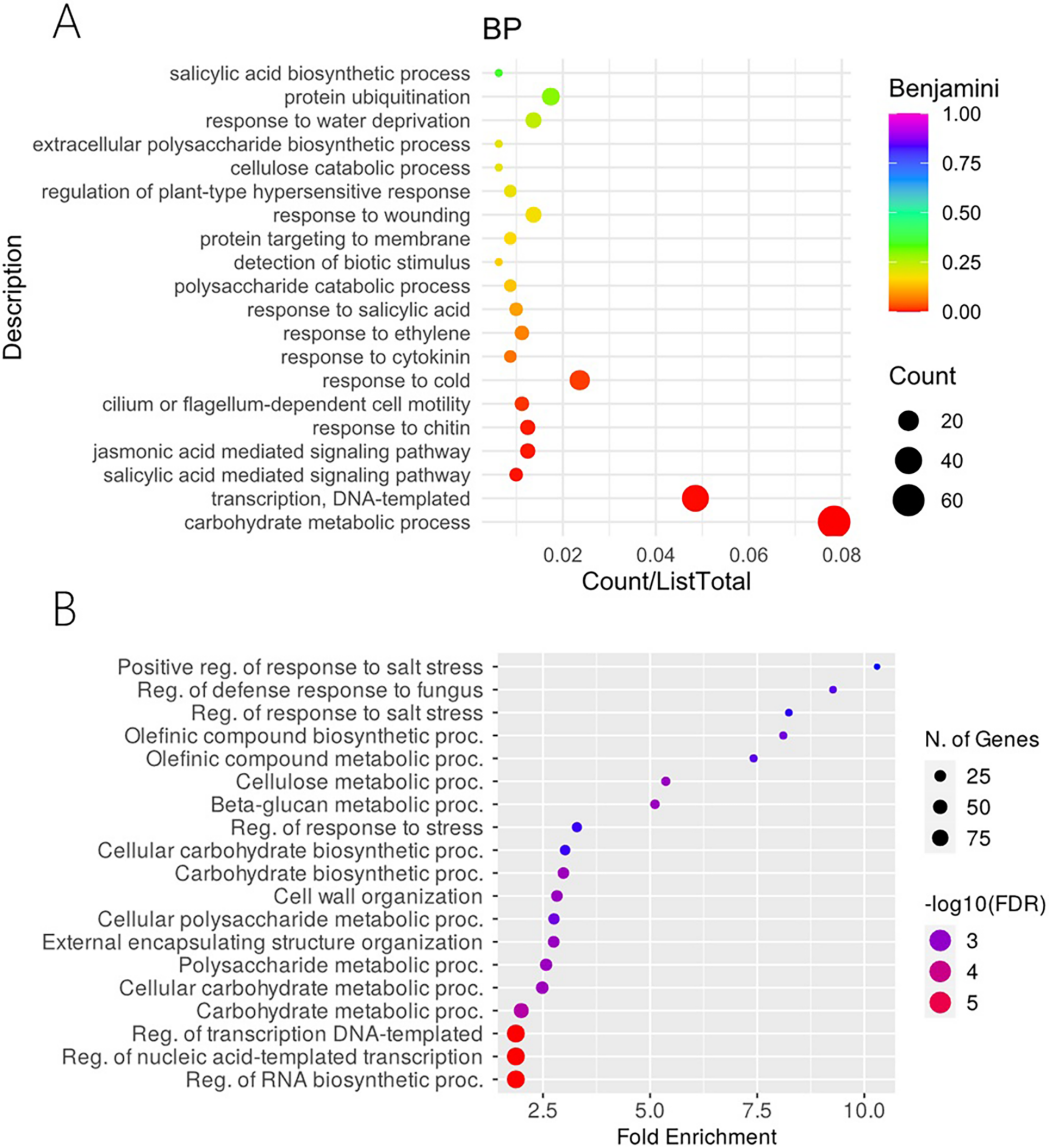
Differentially expressed genes linked to specific biological functions in R + A vs. R samples. R indicates rice grown by itself, while R + A refers to rice plants grown in co‐cultivation with azolla. Several Gene Ontology terms of the Biological Process category related to carbohydrate and cellulose metabolism, transcriptional regulation, and stress are enriched. (A) Functional enrichment analysis performed in CARMO; (B) Functional enrichment analysis performed in ShinyGO.

We compared the RNA‐seq datasets from inflorescences of R and R + A plants during the stages when primary branch meristems (PBMs) and secondary branch meristems (SBMs) developed. We identified 997 differentially expressed genes, of which 797 were upregulated and 200 downregulated in the R + A samples. Among these, several are related to carbohydrate and cellulose metabolism, as highlighted by the functional enrichment analysis performed both with ShinyGO and CARMO (Figure 6). Several categories of stress‐related pathways, both biotic and abiotic, were enriched, as well as jasmonic and salicylic acid signalling pathways, though the latter two were only found when using the CARMO analysis. Moreover, 115 transcription factor encoding genes belonging primarily to the *APETALA2/Ethylene Responsive Factor* (*AP2/ERF*; 29 genes, of which 28 were upregulated), *WRKY* (10 genes, all up‐regulated) and No Apical Meristem (NAM; 9 genes, all up‐regulated) gene families (Figure S5, Tables 1–3).

## 4 DISCUSSION

### 4.1 Rice plants growing together with azolla partition more biomass to shoot tissues

The co‐cultivation of rice with azolla and its symbiont has been shown to provide an increase in N nutrition and, thereby rice yield (Kröck et al. 1988; De Macale and Vlek 2004; Bhuvaneshwari and Singh 2015). Nevertheless, aside from a role in N nutrition, it still remains unclear if the presence of azolla during its co‐cultivation with rice could play an additional role in rice physiology and development. In the present study, we observed that under non‐limiting N conditions, the presence of azolla and its symbiont altered the allocation of biomass between roots and shoots compared to rice plants grown alone, resulting in a greater allocation of biomass to shoots during the vegetative phase. This increased allocation of resources to the shoots resulted in a 1.4‐fold increase in leaf number, while the number of tillers remained similar between R and R + A plants. This observed shift in resources allocation between roots and shoots as a result of the presence of azolla in the vegetative stage matches those observed in parallel studies where these changes were ascribed to altered increased availability of phytohormones (which are released by azolla, such as cytokinins, ABA and auxins) and Fe in the medium (Cannavò et al., 2024).

### 4.2 Increased sink capacity and improved panicle traits in R + A plants do not translate into greater yields

To further dissect the impact of azolla on rice during rice‐azolla co‐cultivation, we assessed traits directly associated with grain yield. Notably, the presence of azolla and its symbiotic cyanobacteria in R + A enhanced several traits that improved panicle architecture (Figure 3). In particular, growing rice plants with azolla led to an increase in panicle length and branching, which in turn increased grain numbers per panicle. Several studies have reported a positive relationship between grain yield and the alterations we observed in R + A plants both in the vegetative state (i.e. increased resource allocation to shoots, Zhang et al., 2015) and during the reproductive phase (i.e. panicle architecture, Agata et al. 2020). Surprisingly, however, this greater resource allocation to shoots in the vegetative phase and the improved yield attributes in R + A plants did not translate into greater grain yield compared to R plants. While further studies are certainly required to understand the mechanisms underlying this response, the lack of grain yield improvement in R + A plants might suggest either a reduced partitioning of carbon to reproductive tissues or photosynthetic capacity for the R + A plants not sufficient to fill the greater number of grains in the presence of azolla (thus leading to a larger sink, which is a product of both grain number and size, Nakano et al., 2017). A similar result has recently been reported by researchers that increased sink capacity in grains in rice by cloning the quantitative trait locus for *MORE PANICLES 3* (*MP3*) in near‐isogenic lines (NILs, Takai et al., 2023). As observed in our study, they showed that with an increase in spikelet number and sink capacity in NIL‐*MP3* compared to the parental cultivars, the overall yield did not improve. Further comparative experiments under ambient and elevated CO_2_ concentrations, however, demonstrated that this was likely to be caused by limited photosynthetic capacity at ambient CO_2_ levels, as NIL‐*MP3* yields were significantly greater than those of the parental cultivar Takanari only under elevated CO_2_ conditions (Ohkubo et al. 2020). Furthermore, it will be interesting to repeat in the future the rice‐azolla co‐cultivation system using mutants that have been shown to increase grain size like *grain size 3* (*gs3*, Fan et al. 2006) or *big grain1* (*Bg1‐D*, Liu et al., 2015) to understand if combining rice‐azolla co‐cultivation with favourable genotypes could further enhance rice grain yield.

### 4.3 Co‐cultivation of rice with azolla altered grain mineral concentrations, with positive and negative effects

Being a staple food for half of the world's population, improving the nutritional quality of rice is a critical issue, especially regarding Fe and Zn deficiencies. The general improvement of elemental concentrations in the rice grains indicates that the presence of azolla and its symbiont alter micro and macronutrient uptake at the root level, translocation and/or partitioning between rice tissues or into rice grains. While the experimental setup used does not allow us to determine whether these effects were primarily driven by azolla and one of its symbionts, previous work has shown that inoculation of bacterial and cyanobacterial strains alone can improve elemental concentrations in crop plants as they release different biological active substances (e.g. phytohormones, siderophores) that boost plant nutrient use efficiency and their nutritional profiles (Rana et al. 2015; Nain et al., 2010; Prasanna et al., 2013; Santini et al. 2021). Surprisingly, we observed a decline in the Zn concentration in grains of co‐cultivated rice. Although further studies are needed to clarify the underlying mechanisms, the reported impact of co‐cultivation rice with azolla and its symbiont on increased Fe bioavailability (Cannavò et al., 2024) could explain these reduced Zn grain concentrations, as high Fe is known to impair Zn uptake (Giordano et al. 1974; Khaliq et al. 2019). Indeed, the presence of azolla was recently found to alter the expression patterns of genes involved in Fe absorption, homeostasis, inclusion and sequestration in rice roots, thus suggesting that R + A experienced a condition of Fe excess (Cannavò et al. 2024). Thus, considering that Zn malnutrition has become one of the major public health concerns worldwide and that rice Zn biofortification is considered one of the key strategies to address/mitigate this challenge (Senguttuvel et al. 2023), the results presented here raise important concerns regarding cascading effects on plant nutrient use efficiency and grain quality. Further analyses are needed to understand the physiological mechanisms underlying the azolla‐induced variations in grain ionome, also considering contrasting soils, with different chemistries (e.g. with different Fe levels). This is a fundamental aspect, with important implications for nutritional security, that needs to be addressed to fully understand how azolla‐rice co‐cultivation could contribute to more sustainable rice production systems.

### 4.4 Co‐cultivation of rice with azolla led to the upregulation of numerous transcription factor genes involved in spikelet development and grain size and shape

Panicle architecture and grain morphology differences are partially supported by RNA‐seq data, which show changes in expression in genes related to cellulose metabolism, cell wall synthesis and grain shape. Three cellulose synthase genes were upregulated, as well as five glycosyl transferases. *Oryza Grain Length on Chromosome 7* (*GL7*) (Os07g0603300) was found to be significantly upregulated (log2FC 1.75) in R + A samples. This gene is related to a slender grain shape (Wang et al. 2015), and the experimental data showed a slight elongation of the grain in R + A with respect to the R samples.

A substantial number of transcription factors were upregulated in the presence of azolla, particularly of three families: *AP2/ERF, WRKY* and NAM. These families have been shown to be involved in multiple processes related to stress response, plant growth and development (Feng et al. 2020; Singh et al. 2021; Wani et al. 2021). The *AP2/ERF* (*APETALA2/ethylene‐responsive element binding factors*) family counts 164 members in rice (Xie et al. 2022), and only for a few of them gene functions have been elucidated. Twenty‐four genes belonging to this family were found to be highly upregulated when co‐cultivating with azolla, while only one of them was downregulated (*OsRAV12*). Several *AP2/ERF* genes were found to be involved in spikelet development, and others were shown to be involved in grain size and shape determination by activating starch accumulation, cell elongation and division (Xie et al. 2022). For example, overexpression lines of *OsERF102* (Log2FC: 6.22) showed panicle phenotypes similar to those observed in this study, with increased secondary branching and more grains per panicle (Qi et al. 2011). The 100‐grain weight was also slightly decreased in these overexpression lines.

*OsDREB1C*, belonging to the DREB (Dehydration Responsive Element‐Binding) subfamily of *AP2/ERF* proteins, has been linked to increased nitrogen use efficiency and grain yield (Wei et al. 2022). However, none of the proposed targets of this TF was found to be upregulated in the present study. Factors of the *RAV* (Related to ABI3/VP) subfamily have been proposed as regulators of flowering and carpel development (Osnato et al. 2020); here, we show that *OsRAV11* and *OsRAV12* were found to be respectively upregulated and downregulated in azolla co‐cultivated samples.

NAM (No Apical Meristem) transcription factors are also involved in response to several types of stress and are mediators of secondary cell wall biosynthesis (Singh et al. 2021). Activation of these transcription factor families might have a role in altering the pathways responsible for panicle development and may cause the differences that we observed.

### 4.5 Co‐cultivation with azolla may induce defence priming

Genes of the *AP2/ERF* family are involved in response to several abiotic stresses (Feng et al. 2020). Significant upregulation was detected for the *OsDREB1A*, *1C, 1D, 1E, 1F, 1G* genes. Upregulation of these genes has been shown to improve plant resistance to drought, salt and cold stress (Xie et al. 2022). The activation of these genes induced by azolla co‐cultivation might prime the plant to face future stresses better. Indeed, responses to various types of stresses were significantly enriched pathways according to the Gene Ontology analysis. Furthermore, salicylic (SA) and jasmonic acid (JA) signalling pathways were also enriched in the CARMO analysis, with 8 and 10 DEGs, respectively. SA and JA signalling pathways are important for defence responses. *OsMAPK5* and *OsGRX6* are annotated on both pathways, encoding for a protein kinase and a glutaredoxin, respectively.

*WRKY* transcription factors have also been shown to be involved in resistance to several pathogens and abiotic stresses, and several *WRKY* factors were upregulated in azolla co‐cultivated samples. Among these, *OsWRKY71* and *OsWRKY45* encode for regulators of systemic acquired resistance (SAR) and are involved in GA‐ and ABA‐related signalling pathways (Wani et al., 2021). WRKYs are involved in complex regulatory networks, and opposite roles have been reported for the same gene, such as *OsWRKY45* and *OsWRKY76*. Peng et al. (2010) reported that the overexpression of 4 of the *OsWRKYIIa* subfamily members (*OsWRKY62, OsWRKY28, OsWRKY71, OsWRKY76*) led to increased disease resistance.

Considering the upregulation of many genes involved in resistance to stresses, it will be interesting to explore in the future if azolla‐grown rice has an increased ability to respond to biotic and abiotic stresses, such as salt and heat stress, or infection by parasites.

## 5 CONCLUSIONS

The benefits of azolla‐rice co‐cultivation in terms of N nutrition are well recognised. Our study provides evidence that azolla‐rice co‐cultivation could potentially have a positive effect on rice performance aside from N nutrition. Indeed, in non‐limiting N conditions, the presence of azolla improved shoot biomass in the vegetative stage and improved panicle architecture and concentration of several elements in the rice grains in the reproductive stage. Furthermore, our transcriptome datasets suggest a possible role in priming rice plants to abiotic and biotic stresses that deserve further attention given the current climate scenario and the need to increase yield using sustainable agriculture approaches. However, despite these improvements, rice co‐cultivation with azolla did not lead to greater yields compared to the rice grown alone. While the underlying reason for this outcome remains unclear, this highlights the need for further investigation into the effects of azolla on rice performance across a wider range of environmental conditions, including soils with different levels of Fe and other trace metals. This is because the observed azolla‐induced reductions in Zn concentrations in the grains raise concerns about potential cascading effects on grain quality, which could constrain the broader applicability of azolla co‐cultivation practices and warrant further detailed exploration.

## Supporting information


**Data S1:** Supporting Information.

## Data Availability

The data that support the findings of this study are available on reasonable request from the corresponding author.
