# Supplementary material for: Azolla mediated alterations in grain yield and quality in Rice
Source: Physiol Plant. 2025 Mar 26;177(2):e70158. doi: 10.1111/ppl.70158 (PMC11947517; doi:10.1111/ppl.70158)
Supplement: Supplementary file 1 — Data S1: Supporting Information. [file PPL-177-e70158-s001.pdf]

Azolla mediated alterations in grain yield and quality in Rice

Nadia Bazihizina[a], Chiara Paleni[b], Stefania Caparrotta[c], Tania Macchiavelli[c], Giorgia Guardigli[a], Ilaria Colzi[a], Michele Petrillo[c], Cristina Gonnelli[a], Antonietta Saccomanno[b], Veronica Gregis[b], Stefano Mancuso[c], Diego Comparini[c]*, Martin M. Kater[b*], Camilla Pandolfi[c]

a- Department of Biology, Università degli Studi di Firenze, Via Micheli 1, 50121 Florence, Italy
b- Department of Biosciences, Università degli Studi di Milano, Via Celoria 26, 20133 Milan, Italy
c- Department of Agriculture, Food, Environment and Forestry, Università degli Studi di Firenze, Viale delle Idee 30, Sesto Fiorentino, 50019, Italy

* Correspondence
Diego Comparini
E-mail: diego.comparini@unifi.it

Supplementary materials

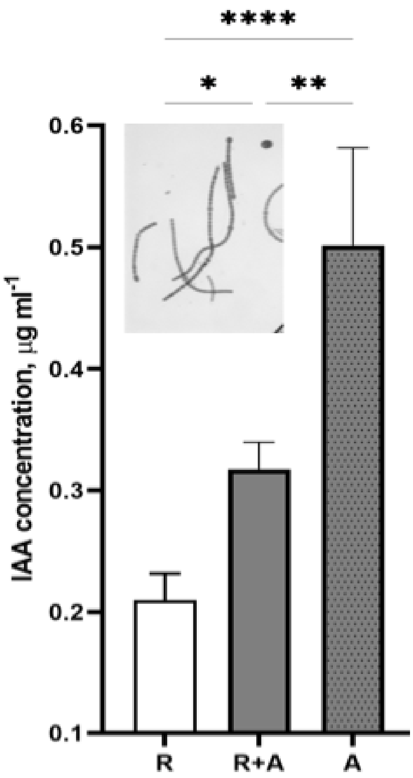

Figure S1. Bar charts illustrating how the concentrations of Indole-3-acetic acid differ among treatments. R indicates rice grown by itself, R+A refers to the azolla-rice co-cultivation and with azolla growing alone (A). The photo shows the cyanobionts found in the leaves of azolla grown in Nitrogen-rich growing solution Yoshida. Asterisks denote significance levels from ANOVA analysis: * $p < 0.05$, ** $p < 0.01$, *** $p < 0.001$, **** $p < 0.0001$. n=3

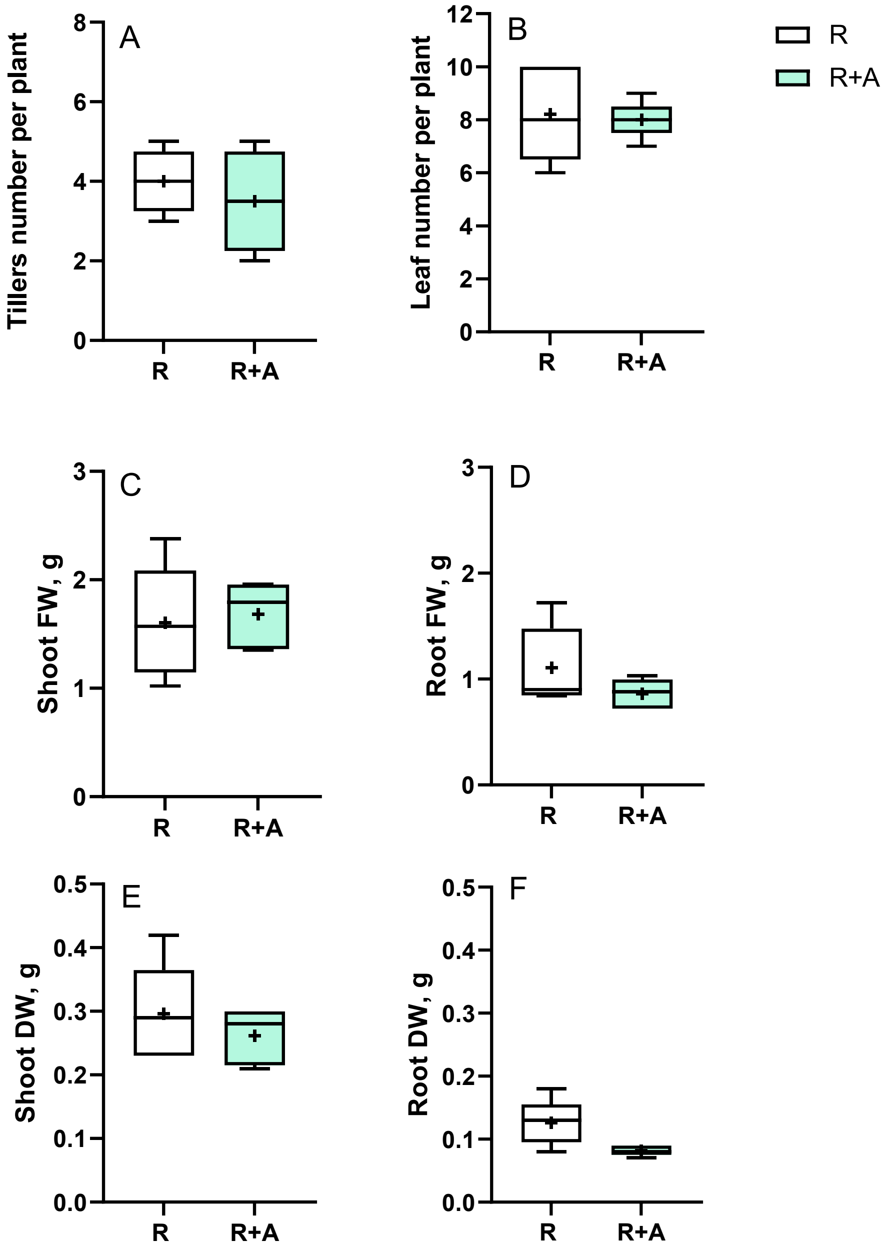

Fig S2: Boxplot graphs showing the number of tillers (A) and leaves (B) per plant, shoot and root fresh (C and D, for shoot and root tissues respectively) and dry weight (E and F, for shoot and root tissues respectively) in rice plants grown alone (R) or in co-cultivation with azolla (R+A) for 30 days. R indicates rice grown by itself while R+A refers to the azolla-rice co-cultivation.

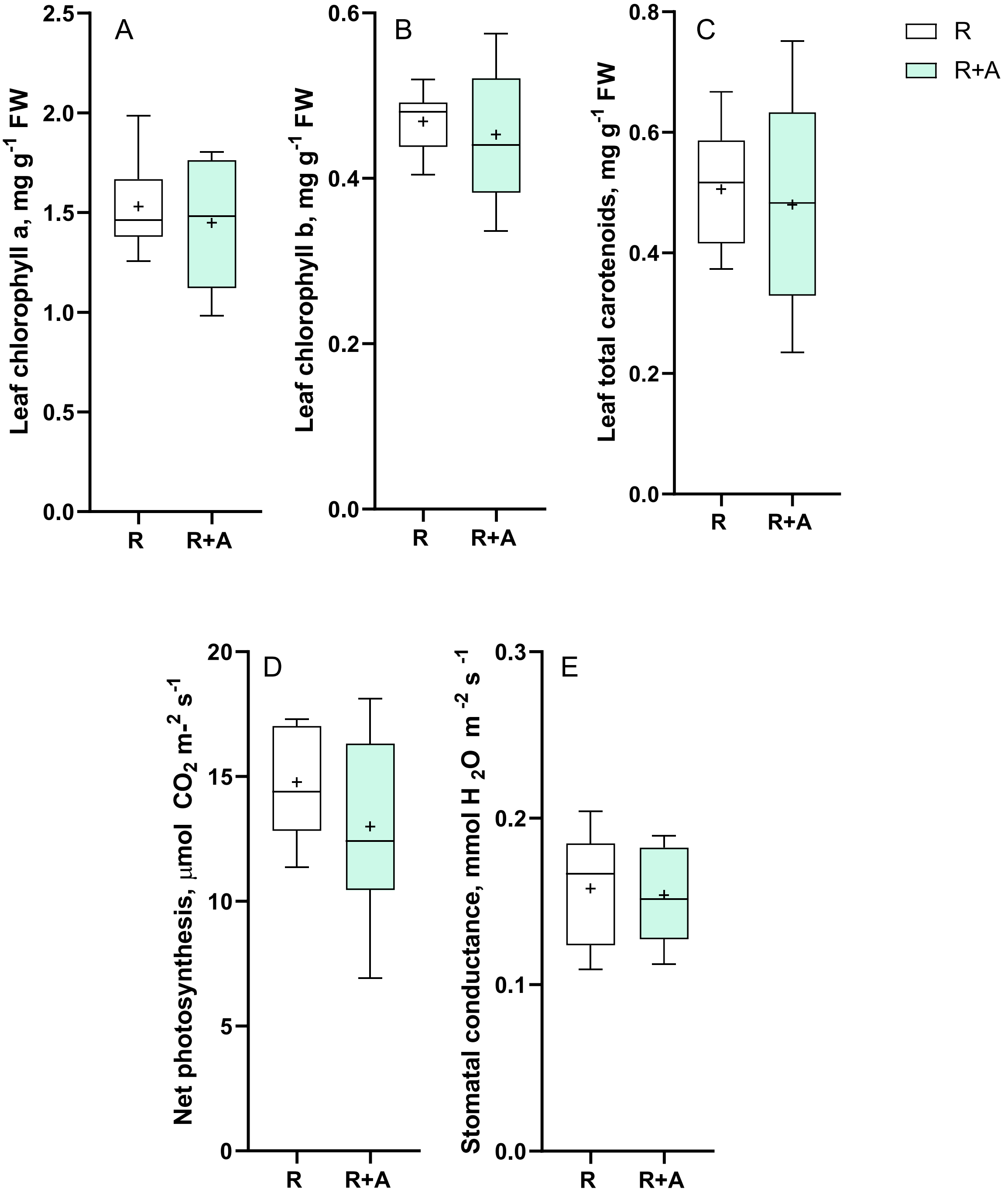

Figure S3. Boxplot graphs illustrating the effect of azolla on leaf photosynthetic pigments and leaf gas exchange parameters of rice plants. A) Concentration of chlorophyll *a* in fresh leaves; B) Concentration of chlorophyll *b* in fresh leaves; C) Concentration of total carotenoids in fresh leaves; D) Net photosynthetic rate; E) Stomatal conductance. R indicates rice grown by itself while R+A refers to the azolla-rice co-cultivation. Asterisks denote significance levels from ANOVA analysis: * $p < 0.05$, ** $p < 0.01$, *** $p < 0.001$. n=8

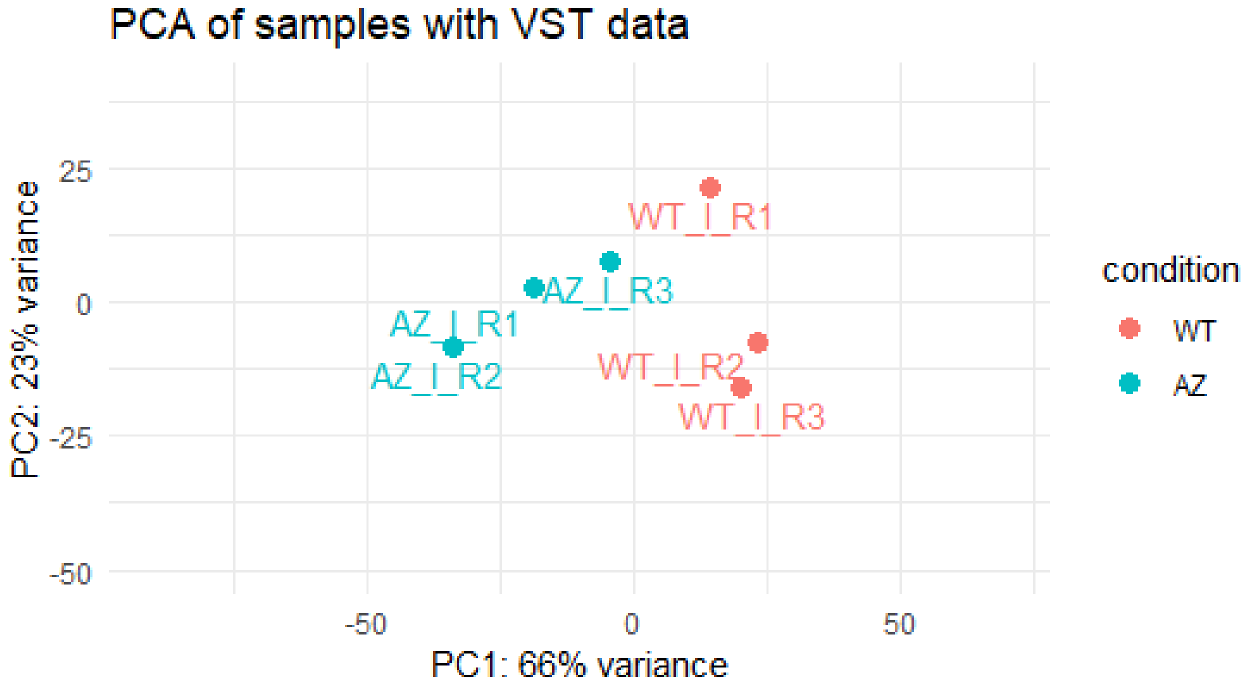

Figure S4. PCA of the VST-transformed RNA-seq data. WT: not co-cultivated with azolla. AZ: co-cultivated with azolla.

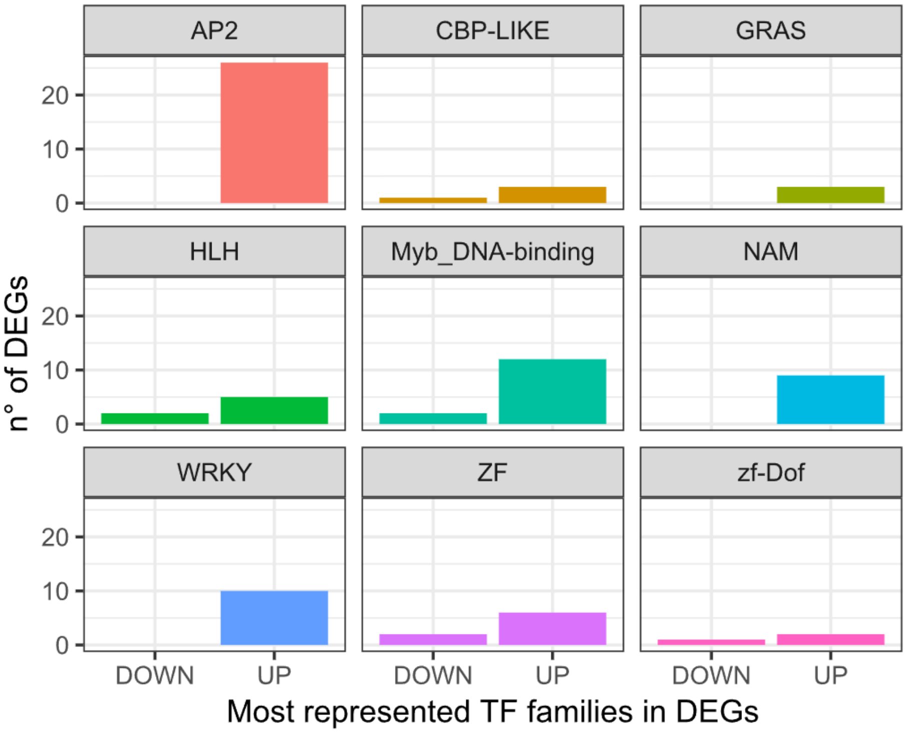

Figure S5. Families of transcription factor encoding genes up- or down-regulated in R+A vs R. The number of either down- or upregulated genes is shown for the most represented transcription factor families in the list of DEGs (n° genes > 2).

Locus ID	Log2FC	padj	Gene name(s)	Synonyms
**Os01g0797600**	1.47	0.002606	OsERF3	OsERF75, AP2/EREBP4, OsBIERF2, OsDLN3
**Os03g0263000**	1.64	0.023968	OsERF9	AP2/EREBP135
**Os06g0196700**	1.15	4.87E-04	OsARF16	
**Os02g0676800**	3.36	6.47E-08	OsERF20	AP2/EREBP126, OsDREB1G
**Os04g0572200**	3.3	0.010125	OsERF23	AP2/EREBP144
**Os02g0656600**	0.83	0.02366	OsERF32	AP/EREBP21, OsDREB2B
**Os04g0549700**	2.62	1.50E-05	OsERF33	OsAP2/ERF40, AP2/EREBP40, OsDREB4-2
**Os04g0550200**	2.02	0.000202	OsERF34	AP2/EREBP42
**Os10g0560700**	0.87	0.000218	OsERF36	AP2/EREBP7
**Os04g0549800**	4.98	8.81E-09	OsERF37	AP2/EREBP41
**Os03g0191900**	1.75	0.000362	OsERF47	AP2/EREBP121
**Os08g0408500**	1.51	0.00012	OsERF48	OsDRAP1, AP2/EREBP170
**Os09g0369000**	0.99	0.010747	OsERF50	OsDREB6, AP2/EREBP113
**Os07g0674800**	4.5	0.000599	OsERF67	AP2/EREBP76
**Os05g0497300**	1.49	0.001391	OsERF74	AP2/EREBP4, OsBIERF2, OsDLN3
**Os04g0610400**	3.05	0.011598	OsERF77	AP2/EREBP 149, OsDLN121
**Os02g0654700**	2.47	8.62E-08	OsERF91	OsAP59, AP2/EREBP147, OsBIERF3
**Os09g0457900**	6.22	0.007271	OsERF102	AP2/EREBP123, OsEATB
**Os02g0764700**	5.44	0.000107	OsERF103	OsDERF5, OsERF109, AP2/EREBP130
**Os06g0553700**	1.37	0.008531	OsERF122	AP2/EREBP56
**Os02g0638650**	2.24	0.000473	OsERF141	AP2/EREBP109
**Os02g0767800**	2.34	0.006627	AP2/EREBP139	
**Os09g0522200**	3.06	0.004379	OsDREB1A	OsERF24, AP2/EREBP114, OsCBF3
**Os06g0127100**	6.44	0.004258	OsDREB1C	OsERF26, AP2/EREBP98, OsCBF2
**Os04g0572400**	4.9	0.006309	OsDREB1E	OsERF30, AP2/EREBP160
**Os01g0968800**	4.36	0.035221	OsDREB1F	OsERF27, AP2/EREBP81
**Os02g0677300**	5.14	0.039963	OsDREB1G	OsERF25, AP2/EREBP138
**Os01g0693400**	1.43	0.004386	OsRAV11	AP2/EREBP127
**Os05g0549800**	-1.81	0.032939	OsRAV12	AP2/EREBP96

Tab S1. List of AP2/ERF transcription factors differentially expressed in the azolla-grown samples.

Locus ID	Log2FC	padj	Gene name(s)	Synonyms
**Os04g0508400**	5.26	4.7351E-10		
**Os03g0815100**	2.23	7.243E-06	SNAC1	OsNAC9, OsNAC19
**Os07g0225300**	1.17	0.02259884	NAC3	OsNAC3, OsDLN180
**Os01g0261200**	0.95	0.02974326	NAC8	OsNTL3, OsNAC8
**Os01g0675800**	2.07	0.00051868	NAC14	OsNAC14
**Os03g0133000**	2.17	0.00028905	NAC22	
**Os10g0359500**	0.72	0.04135597	NAC61	
**Os04g0536500**	2.00	0.00183051	NAC82	OsSWN6
**Os05g0421600**	3.12	0.02532471	NAC88	OsNAC52

Tab S2. List of NAC transcription factors differentially expressed in the azolla-grown samples.

Locus ID	Log2FC	padj	Gene name
**Os01g0246700**	3.33	0.00253938	OsWRKY1
**Os05g0537100**	2.27	0.03182277	OsWRKY7
**Os05g0571200**	1.69	0.01000141	OsWRKY19
**Os01g0821600**	2.86	2.35E-07	OsWRKY21
**Os01g0826400**	2.58	0.00422976	OsWRKY24
**Os05g0322900**	1.51	0.00053148	OsWRKY45
**Os02g0181300**	3.56	0.01817818	OsWRKY71
**Os09g0334500**	2.42	0.02561687	OsWRKY74
**Os09g0417600**	3.41	0.00969667	OsWRKY76
**Os03g0855100**	1.13	0.00486725	OsWRKY80

Tab S3. List of WRKY transcription factors differentially expressed in the azolla-grown samples.
